# The Usefulness of Edible and Medicinal Fabaceae in Argentine and Chilean Patagonia: Environmental Availability and Other Sources of Supply

**DOI:** 10.1155/2012/901918

**Published:** 2011-12-13

**Authors:** Soledad Molares, Ana Ladio

**Affiliations:** INIBIOMA, Universidad Nacional del Comahue-CONICET, Quintral 1250, Bariloche, Río Negro 8400, Argentina

## Abstract

Fabaceae is of great ethnobotanical importance in indigenous and urban communities throughout the world. This work presents a revision of the use of Fabaceae as a food and/or medicinal resource in Argentine-Chilean Patagonia. It is based on a bibliographical analysis of 27 ethnobotanical sources and catalogues of regional flora. Approximately 234 wild species grow in Patagonia, mainly (60%) in arid environments, whilst the remainder belong to Sub-Antarctic forest. It was found that 12.8% (30 species), mainly woody, conspicuous plants, are collected for food or medicines. Most of the species used grow in arid environments. Cultivation and purchase/barter enrich the Fabaceae offer, bringing it up to a total of 63 species. The richness of native and exotic species, and the existence of multiple strategies for obtaining these plants, indicates hybridization of knowledge and practices. Only 22% of the total species used are mentioned in bothcontexts of food and medicine, reflecting low-use complementation. This study suggests a significant ecological appearance and a high level of availability in shops and exchange networks in Patagonia, highlighting the need to consider the full set of environmental and socioeconomic factors in research related to the use and cultural importance of plants in regional contexts.

## 1. Introduction

Fabaceae is a family of cosmopolitan distribution, with approximately 730 genera and 19.400 species, lying in third place after Asteraceae and Orchidaceae with respect to species richness at a global level [1]. This high species richness is reflected in great morphological and chemical diversity, from which multiple uses are derived [2]. In socioeconomic terms, their importance for health and human alimentation is highlighted, although they also provide wood resources and dyes, resins, insecticides, fibres, fodder, and so forth [3, 4].

The nutritional value of Fabaceae is to a great extent due to their ability to fix atmospheric nitrogen for protein synthesis. This advantage has led to protein concentrations in leaves and seeds which vary between 20% and 40% dry weight, depending on the species [5]. Human societies have learned to select and incorporate mainly the seeds and fruit into their diet, taking advantage of the variety of essential amino acids they contain. In various cultures around the world they have become the principal providers of nonanimal protein, accompanying the carbohydrates provided by cereals [4].

Proof of their importance in human nourishment is the fact that they are, along with cereals, amongst the first plants to have been domesticated [5]. In America the cultivation of Fabaceae dates from prehistoric times [6, 7]. For example, at least five species of the genus *Phaseolus *(beans) were found in a wide range of geographic zones, from the meridional Andes to Mesoamerica and the Caribbean [8], very often along with *Zea mays* (“maize” and “corn”) [4].

In Argentine Patagonia, Fabaceae seems to be one of the main gathered wild food resources, both in multiethnic populations situated in the Sub-Antarctic forest [9] and in indigenous communities of the arid steppe and Patagonian monte environments [10]. It is suggested by Pardo and Pizarro [11] that during the first stages of the Spanish colonization, the introduction of “chickpeas” (*Cicer arietinum), *“peas” (*Pisum sativum*), and “broad beans” (*Vicia faba*) from Europe to this part of the continent must have been extremely successful. The current importance of these exotic legumes is well known, since in many Patagonian vegetable gardens “broad beans”and “peas”are still cultivated as the main dietary resource [12].

With regard to medicinal uses, it has been pointed out that they are found amongst the five botanical families richest in therapeutic properties in the pharmacopeia of indigenous and rural populations in Holarctic [13], Neotropical [14], and Sub-Antarctic [15] regions. In particular, Barboza et al. [16] found that in Argentina Fabaceae is second in importance to Asteraceae in terms of richness of medicinal taxa. Their medicinal value lies partly in their effectiveness in the treatment of a wide variety of human ailments [17]. The variety of chemically active constituents, such as tannins, flavonoids, alkaloids, and terpenes often found in members of this family, are substances with a high level of biological activity, and the fact that they are used extensively would suggest a pattern of global ethnomedical knowledge [13, 18]. However, other ecological, morphological, and sociocultural factors in addition to these chemical-nutritional ones may explain more fully the vast use made of this botanical family [15, 19]. For example, the presence of coloured and/or conspicuous aerial organs, notable organoleptic features, and their diversity in local flora are characteristics which would probably have attracted the attention of human populations, leading to experimentation and use [14, 20, 21].

Ethnobotanical theory has widely indicated the importance of the environment as a determining factor in the selection of useful resources by human populations [22–25]. The work of Phillips and Gentry [26] was the first to make the connection in tropical and subtropical populations between higher density and abundance of plant species with greater cultural importance to the people. Later, the ideas of the ecological appearance theory [27, 28], applied to ethnobotanical studies, have been very useful in determining whether plant-human interaction is influenced by the ecological and chemical characteristics of the plants [29]. Following this idea, humans would prefer to forage for plants which are most visible (the most abundant species, large in size and/or perennial) for their food, also taking advantage of the benefits of their antiherbivore defences for medicinal resources [29, 30]. This approach assumes a direct relationship between species availability in any given environment and its cultural importance. From this perspective, people will have more opportunities to see and learn about the species growing closest to their dwellings and most available in time and space, than those which are less common, short lasting, or inaccessible [29, 31, 32].

Having said this, however, in current human populations (even the most isolated) people do not only select and depend on the resources available in their immediate ecological surroundings. The appearance or availability of plants may also be enriched and strongly influenced by cultural exchange processes, barter, and/or socioeconomic practices that enable a culture to use a distant resource, or one not belonging to their environment [33–35]. In addition, the socioeconomic relevance of certain resources of global importance [36] and their diffusion by the communications media have, since ancient times, moulded dietary habits and practices associated with health [37, 38]. Other supply sources, therefore, must be considered in the evaluation of the cultural importance of plant species.

Another point which has been linked to the cultural relevance of plants is their varied use in multiple contexts of alimentation and the healing of ailments, indicating a remarkable level of exploration and use intensification due to their singular, specific qualities [39–41]. The use of species considered to be functional foods or nutraceuticals follows a diffuse gradient which includes medicinal function, alimentary, or mixed, depending on the consumer's circumstances. Their complementary, multiple uses seem to be a characteristic of traditional and/or rural societies that maintain strong bonds with their environment [39, 40, 42]. However, in modern communities, which have more contact with market societies, the importance of these resources may vary or change, taking on new significance or value [43, 44].

The object of this study, then, is to ascertain the cultural importance and use of the Fabaceae family as a food and/or medical resource in Argentine-Chilean Patagonia. Using an approach which is based on the importance of the “ecological and nonecological appearance”, of plants to humans, and bibliographical analysis, we will try to understand the current use of this plant group, considering this to be an indicator of its ethnobotanical relevance at a regional level. The preliminary questions we posed were as follows. (1) What does the environment have to offer in terms of Fabaceae species richness in the different ecological environments of Patagonia, and what is the cultural importance of these wild species as medicines and/or food to the local population? (2) Are the wild Fabaceae selected as food and/or medicine the most visible species (mostly shrubs or trees)? (3) Given their historical socioeconomic importance, are there other cultural ways of obtaining Fabaceae used by the current population? (4) Are the Fabaceae chosen for nutritional and therapeutic purposes used in a complementary way? (5) What is the current regional pattern of Fabaceae use by Patagonian dwellers?

## 2. Methods

### 2.1. Study Area

**Figure 1:**
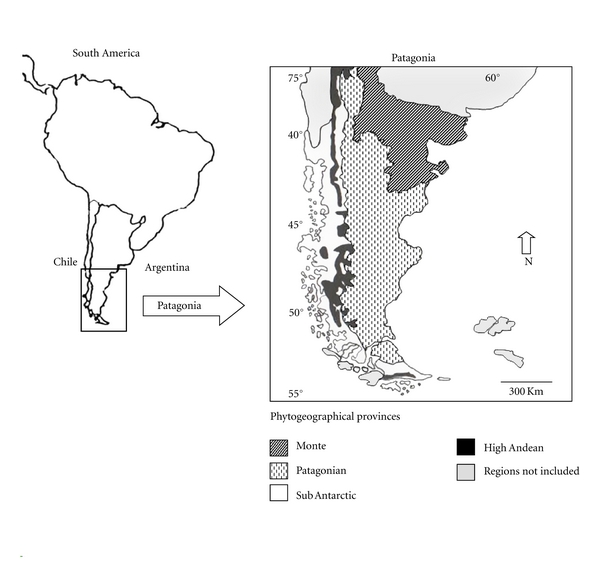
Study map of the Patagonian region and their phytogeographic provinces.

The geographical area studied lies between 37° S and 46° S and includes ecological communities established in different phytogeographical provinces: Patagonian, Monte, and Sub-Antarctic (Figure 1). The Patagonian and Monte provinces (Argentina) extend over plateaus and low mountains in cold, dry climates with annual precipitation varying between 100 and 270 mm. The dominant Patagonian vegetation is grass-steppe or shrub-steppe with scrub and/or cushion species. The Monte vegetation is xerophytic, psammophytic, or halophytic scrub. The Sub-Antarctic province (Argentina and Chile) extends across mountains and glacial valleys, where the climate is temperate and humid, and annual precipitations can reach 2000 mm. The environment varies; there may be deciduous or evergreen forest with patches of grassland and peat bogs, amongst other possible vegetation types [45]. This phytogeographical heterogeneity leads to a high level of diversity in environments, microhabitats, life forms, biological associations, and plant species used by local populations [21].

The ethnobotanical articles analysed are from studies carried out in Chilean and Argentine Patagonia, mainly in small rural communities, of varying cultural origins (Mapuche, Creole, and/or Selk'nam). One urban community was also studied (San Carlos de Bariloche). In general, the rural communities of the indigenous peoples and Creoles studied were found on marginal lands, often in arid regions. Their economy is based on the breeding of domestic livestock. With the various political processes that brought about a restructuring of their lifestyle [46], they began a process of assimilation of the dominant societies, leading to migration of the younger generations to the urban centres, and an associated impoverishment of their living conditions. Despite all this, their culture of a strong connection with the earth and its natural resources is still alive, as are their practices of plant, animal, and mineral gathering. The urban population of Bariloche, in contrast, is highly heterogeneous and multiethnic in character [47].

### 2.2. Bibliographical Analysis

The environmental availability of Fabaceae in terms of species richness present in the flora of Argentine and Chilean Patagonia was analysed using Correa [48] and the Catalogue of Vascular Plants of Southern South America [49] as sources. A value for total richness of the area was obtained, as well as the relative richness of the Sub-Antarctic, Patagonian, and Monte phytogeographical provinces. The richness of Fabaceae used for food and medicinal purposes in the region was estimated from the establishment of a database compiled from ethnobotanical studies. Of approximately 50 studies reviewed, 27 sources were selected which mentioned at least one Fabaceae species in connection with the uses of interest to us. These sources included Biology graduates' degree theses, reviews, and scientific articles. The majority of these refer to the gathering and use of wild plants in rural areas on the part of Mapuche or Creole populations [10, 20, 25, 35, 50–64]. Five studies are review works [9, 65–68]. Few works make reference to the urban ethnobotany of Patagonian cities [69, 70] or deal with the cultural practices of other indigenous Patagonian populations such as the Selk'nam [71], revealing an imbalance in the topics studied in this region.

It should be mentioned that the principal variable studied in this work was Fabaceae species richness, not considering the frequency of cites per species, since this variable was not analysed in most of the works reviewed. This approach assumes that the accumulated richness of all the studies is a reflection of the level of cultural importance of the family under consideration. The heterogeneity of the studies due to the different environments dealt with and/or differences in sociocultural backgrounds was not analysed in this work, since our aim was to explore the role of Fabaceae on a purely utilitarian scale, in a wide sense.

### 2.3. Data Analysis

As an indicator of the cultural importance of the use of Fabaceae, we used the accumulated number of medicinal, edible, or mixed-use species in all 27 publications analysed. All species were categorised according to (1) biogeographical origin: native or exotic plants growing in Patagonia, and (2) methods of obtaining the plants: gathering, cultivation, and purchase-barter cited in each work. In addition, from the information obtained from the Catalogues of Patagonian Flora [48], of Vascular Plants from Southern South America [49], and from our own registers, the wild species obtained through gathering were categorised according to (3) ecological environment of origin: forest (Sub-Antarctic forests), steppe (Patagonian province), and/or monte, and (4) life form: herbs, shrubs, and trees.

The ecological availability of wild Fabaceae and species richness for medicinal and/or edible purposes corresponding to the three phytogeographical Patagonian environments (Sub-Antarctic, Patagonian, and Monte), their life forms (herbs, shrubs, and trees), and their supply strategies were compared with the *χ* ^2^ test (*P* < .05). Biogeographical origin, native or exotic, of the wild medicinal and/or edible species for each environment was compared using the binomial test (*P* < .05). The similarity of wild Fabaceae in these different environments was analysed with the Jaccard index (JI:* c*/(*a *+* b *+* c*) × 100), where *c *is the number of plants that two environments have in common, *a* is the number of species unique to one environment, and *b *is the number of species unique to the other one [72]. With the objective of describing the current use pattern of Fabaceae by means of an integrated view of the relationships between all the variables analysed (biogeographic origin, use, life form, supply strategy), MDS (Multidimensional scale analysis) was used. MDS provides a spatial representation of the data that shows the positions of all the variables relative to each other [73]. The proportion of variation explained by this representation was measured with the value of *R* ^2^ (which varies between 0 and 1) and the stress, which is a measure of fit of the distances created, whose values for a good fit should be less than 0.1 [73].

## 3. Results and Discussion

### 3.1. The Ecological Environments of Patagonia: The Environmental Availability and Cultural Importance of Fabaceae

**Figure 2:**
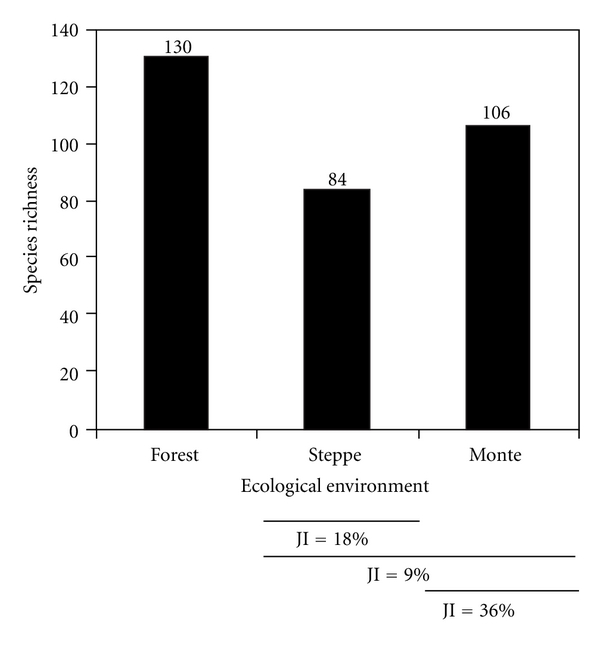
Wild Fabaceae richness in Argentine and Chilean Patagonia according to Correa [48] and Zuloaga et al. [49]. The JI values represent the percentage of common species among environments.

Argentine-Chilean Patagonia offers a diverse selection of wild Fabaceae, estimated at some 24 genera with 234 species and varieties. The Andean-Patagonian forests of Argentina and Chile present higher species richness than the arid provinces of the Argentine steppe and monte (forest: 130 species (40.6%), monte: 106 species (33.12%), and steppe: 84 species (26.25%); *χ* ^2^ = 9,9; df = 2; *P* < .05, Figure 2). However, taken together (steppe plus monte), almost 60% of the species come from the arid Patagonian regions of Argentina, showing the significant preponderance of this group in these phytogeographic environments. The low similarity indices indicate that each of the ecological environments offers a set of unique species, especially the most humid forest region and the arid zones (JI = 9%), although the monte and steppe environments have a higher species coincidence (JI = 36%), when compared (Figure 2).

 According to the bibliographical survey, local populations have identified and learned about the use of Fabaceae, including them in their daily lives as food and/or medicine. Of the 234 species that grow in Patagonia, some 30 species are used (12.8% of the total). Nineteen of these species are medicinal and 17 are edible (8% and 7% of the total flora, resp.) (Table 1). In relation to this, Rapoport and Drausal [38], in a study comparing flora from different regions of the planet, have suggested that for any environment or biota, a minimum of 10% of species can be expected to be edible. Our results, therefore, may mean that the listing obtained in this study is not exhaustive and does not reflect the complete richness of useful resources for humans, something which can be surveyed in future ethnobotanical studies.

**Figure 3:**
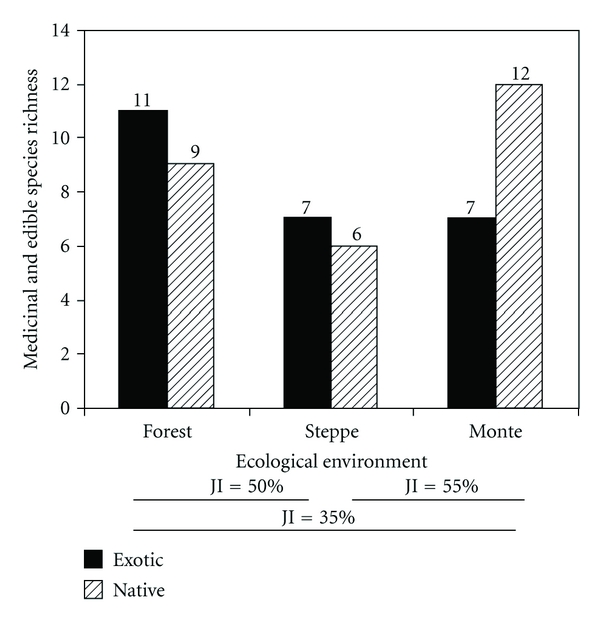
Edible and medicinal species richness of Fabaceae cited by 27 ethnobotanical sources and their ecological environments in Patagonia. JI: Jaccard similarity index.

The bibliographical sources studied indicate that the proportion of medicinal and/or edible species used do not show any preference towards species from the forest (20 species: 66%), from the monte (19 species: 63%), or the steppe (13 species: 43%) (*χ* ^2^ = 1,6; df = 2; *P* > .05.  Figure 3). However, the arid zones (monte and steppe) contribute significantly more medicinal and/or edible species when taken together (Binomial test, *P* < .05). These results may reflect the high proportion of Fabaceae used which come from arid zones, and also the cultural importance of the forests, reflecting their greater structural complexity and plant richness in comparison with the other environments.

The notable advantage taken of arid zones for obtaining Fabaceae should also be analysed within the historical and political context of Patagonian communities. These populations, particularly those of indigenous ancestry, were originally from the subantarctic forests of Argentina and Chile [51]. Due to various sociopolitical processes [46] at the end of the 19th and beginning of the 20th century, these communities were forced to settle in areas on the edges of forests, that is, in the driest zones of Argentina, where they still live today [15]. The forests were either sold or became national and provincial biological reserves, inaccessible in terms of plant and animal resources [15]. This drastic change has very probably influenced socioenvironmental perception of Patagonian environments, affecting behaviour and the differential evaluation of alimentary and medicinal resources [74], and favoring the incorporation of the most available species in the new area settled [74]. De Lucena et al. [75] suggest that in populations which are constantly on the move, where access to resources within protected areas is impeded in some way, and in which the younger generations show little interest in learning about natural resources, the used value of the species would be the result of the people's capacity to adapt to the specific environmental conditions, including learning about the use of the species which are most available at that particular time.

Fabaceae is a botanical family of great importance amongst the flora of the world; many studies have revealed its abundance and ecological and cultural importance in arid regions of the planet, such as Brazil [75, 76], México [77], Africa [74, 78, 79]. Our preliminary results, with communities from arid environments well represented in our investigation, appear to show the same tendencies.

The Fabaceae species used as medicine or food in the region show much more similarity between environments (JI = 35%–JI = 55%.  Figure 3) than the species registered in the published floras (Figure 2). In other words, many useful species are shared between environments, which is directly connected to the high proportion of exotic Fabaceae, principally used for medicinal purposes, which are repeated along the environmental gradient and are not documented in the regional flora. These findings are indicative of the importance of ethnobotanical studies as points of reference and for the updating of regional flora and also point out the need for continuous feedback.

Parallel to this, it was found that the proportion of species of native and exotic origin used by locals in each environment was similar (binomial test_forest,steppe,monte_, *P* > .05), indicating a high degree of incorporation of exotic resources. In general, exotic species have been widely spread and have adapted to all Patagonian Cultural landscapes [15, 21]. The high level of richness of these species seems to be associated with the growing abundance of anthropic environments in the region [58, 80]. Many exotic species growing in Patagonia have been pioneers in degraded environments and have rapidly become invasive [80]. At the same time these species, which tend to be “r” strategists in ecological succession [81, 82], have a repertory of phytochemicals for antiherbivore defence that are useful for medicinal purposes [24, 82]. In addition, some of them have been introduced from Europe, where for centuries they have enjoyed great prestige as therapeutic and/or edible resources (e.g., [83–85]). Publicity relating to their medicinal qualities is also regularly transmitted throughout the region, by means of media such as books, television, and radio [70]. All of the above suggests that the effective use of representatives of this botanical family is markedly influenced by its prominence both in cultural and in ecological terms.

### 3.2. Life Forms of Medicinal and/or Edible Wild Fabaceae in Patagonia

**Figure 4:**
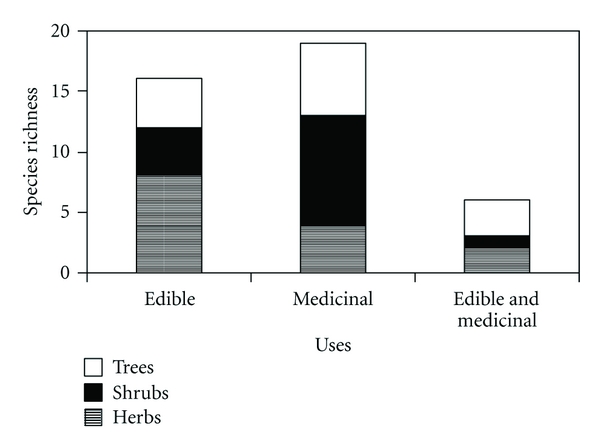
Richness of herbs, shrubs, and trees between wild edible and/or medicinal Fabaceae plants of Patagonia.

Amongst the medicinal, edible, or mixed-use Fabaceae are found herbs, shrubs, and trees (*P* > .05) (Figure 4). However, a high proportion of woody Fabaceae (trees and shrubs) is observed in the three used categories analysed (*P* < .05). The high level of use of wild trees and shrubs for various purposes, including food, has also been highlighted in tropical areas and semiarid regions of the world by Felker [86]. Even though the proportion of herbs is lower in all areas, it is important to mention that in the case of edible use, plant richness with this life form is twice as high as for medicinal plants.

These results are in accordance with the theory of ecological appearance, favouring the greater use of the most conspicuous species in the environments. Similar results were found by Almeida et al. [82] and Albuquerque et al. [29] in studies of medicinal plants carried out in the semiarid region in the Northwest of Brazil, highlighting the high richness of native woody plant species. Our results, however, go against the tendencies found in tropical and subtropical zones, where herbs seem to fulfil an essential role in pharmacopoeias and diets (e.g., [24, 87, 88]). Nevertheless, even though low dominance values have been found for medicinal species of tropical and subtropical regions, these have often been registered as highly abundant in local flora. Abundance is also an expression of ecological appearance, which would increase their accessibility and use [75].

Our work shows that the contribution of Fabaceae to the set of medicinal and/or edible resources in the region is basically woody plants, visible, and conspicuous. Worthy of mention as examples are the shrubs *Adesmia boronioides* and *Cercidium praecox*, both widely recognised as medicinal on both sides of the Cordillera, the genus *Prosopis*, with trees and shrubs of great edible and medicinal value, and *Sophora* spp. with medicinal trees used since ancient times amongst the native peoples of Chilean Patagonia.

### 3.3. Other Methods of Obtaining Fabaceae Species Used by Local Populations

**Figure 5:**
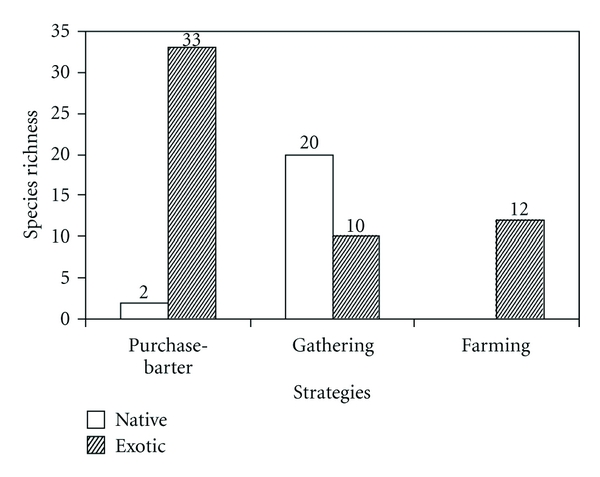
Richness of native and exotic Fabaceae plants obtained by gathering, purchase-barter, and farming in Argentine and Chilean Patagonia.

The medicinal and/or edible species of Fabaceae, as well as being gathered, are also incorporated into the diet and natural and domestic medicine through cultivation, purchase and barter (Figure 5). In this way the richness value rises to a total of 63 species and varieties (more than twice as many), most being of exotic biogeographical origin (43 species) and the remainder native in origin (20) (Table 1).

The environmental supply, then, is increased by the provision of Fabaceae from other biogeographical regions, due to the nutritional, medicinal, and nutraceutical value of these species to Patagonian populations. The highest richness level is reached through the purchase and/or barter of species which are mainly exotic (35 species), followed by the gathering of native and exotic species (30), and finally through the cultivation of exotic species (12) (*χ* ^2^ = 24, df = 2, *P* < .05).

Bartering (e.g., *Caesalpinia paraguarienses, Prosopis rubiflora, Tamarindus indica*) is carried out mainly in rural areas where species from different geographical zones are exchanged by small producers or local livestock breeders, who often travel around as temporary workers at sheep shearing time, a job which allows them to spend time in environments different to their own [89]. The cultivation of Fabaceae species in small vegetable gardens or greenhouses is directly linked with exchange networks between neighbours or through external horticulture or welfare agents, such as the agriculture and fisheries social plan, PSA *(Plan Social Agropecuario) *and Inta (*Instituto Nacional de Tecnología Agropecuaria*) [50]. The purchase of edible Fabaceae in a fresh state (e.g., *Vicia faba, Lens culinaris*), dry or tinned (e.g., *Phaseolus lunatus, P. vulgaris, Cicer arietinum*), takes place in greengrocers, grocers, supermarkets, and whole food stores, whilst those used for medicinal purposes are obtained from herbalists and health food stores (e.g., *Senna alexandrina, S. Stipulacea, *and *Bahuinia forficata*), and this cannot be underestimated. It is worthy of note that *Pisum sativum* and *Vicia faba* are amongst the most sold in the Northwest of Argentine Patagonia, production for both species being estimated at 1625 kg/year just in the area around the city of Bariloche in the province of Río Negro [69].

The resources that form part of the social exchange and barter networks, in general, are considered highly significant in cultural and socioeconomic terms [12]. In this case, purchase and barter reveal that these Fabaceae are resources obtained both internally and externally and are highly valued.

The variety of ways Fabaceae can be obtained might also be a response to the unpredictability of the socioenvironmental contexts of rural Patagonian populations which have been greatly affected by overgrazing and desertification [10]. In these conditions, the appearance and availability of wild resources become highly unstable in time. The diversified strategy could function as a mechanism in subsistence living to maximise the investment of time, energy, and monetary resources in the acquisition of these plants [74].

### 3.4. Complementary Use of Patagonian Fabaceae

Considering all methods of obtaining the plants (gathering, cultivation, purchase, and barter), it was found, although not statistically significant (binomial test, *P* > .05), that this botanical family seems to be more important in Patagonia as a food source (42 species) than as a medicinal resource (35). In agreement with this, Mösbach [62] has pointed out that since ancient times Fabaceae have constituted the basic accompaniment to many traditional recipes in the region, such as stews, jams, and farinaceous preparations.

Only 22% (JI) of the total number of species are used for both food and medicinal purposes (Table 1), reflecting low-use complementation. It has been amply shown that when herbolaria are analysed together, higher complementation is found in the use of medicinal and edible resources in traditional societies, which are distinguished by opposition to the dissociated use generally prevalent in industrialised societies (e.g., [39]). In this region in particular, Ladio [42] found that 63% of the species used in a Mapuche population in the Argentine steppe are used in a complementary way for medicinal and edible purposes. Our results indicate that the Fabaceae family, mainly due to their particular intrinsic characteristics, is used in a more segmented or compartmentalised way.

The great phytochemical and organoleptic diversity that characterises this family of vascular plants is well known [14]. It has been found that the organoleptic perception of many of the secondary metabolites present in the chemical package of this and other groups of plants may be one of the main intercultural clues for the medicinal and/or edible distinction and evaluation of the plants (e.g., [20, 89–91]). Compounds such as alkaloids, isoflavones, coumarins, saponins, and tannins are some of the antinutrients perceived as bitter-tasting which are present in many Fabaceae genera, such as *Astragalus*, *Lupinus, and Melilotus, *and these may orient their consumption towards medicinal use [92].

In response to this detection, and at the same considering the need to take advantage of their high protein content, humans have developed specific cooking and/or detoxification practices, so that species with some level of toxicity can be included in the diet [93–95]. In relation to this, several studies on the alimentation of Patagonian Pre-Columbian populations have documented different techniques of flavour improvement and detoxification using sun-drying followed by the grinding of seeds and edible roots [11]. A deeper study of the phytochemicals and organoleptics of Patagonian Fabaceae could provide the necessary clues to the understanding of this result of low complementation. In addition, it is possible that the higher popularity as a food source of *Phaseolus *spp. and other species in the South American region and in the World in general [79, 84, 86] has contributed to the increase in their use as food in detriment to their use as a medical resource in Patagonia.

### 3.5. An Approach to the Integrated Analysis of the Use of Fabaceae in Patagonia

**Figure 6:**
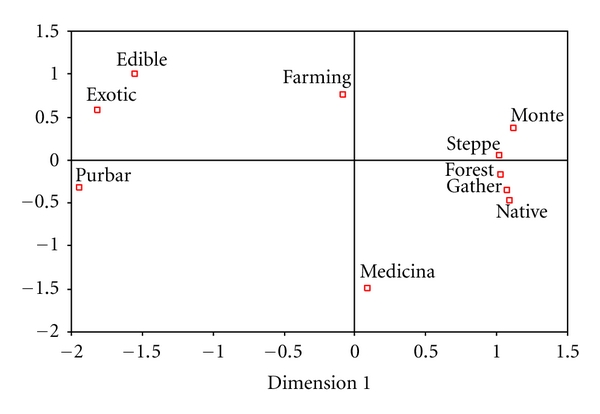
MDS of the data about uses (edible and medicinal), biogeographic origin (native, exotic), strategies (purchase-barter, gathering, farming), and ecological environments (monte, steppe, forest) of the Fabaceae used in Argentine and Chilean Patagonia.

Multidimensional analysis of the data shows a spatial and global representation of the different aspects of Fabaceae use in the region (Stress = 0.07; *R* ^2^ = 0.98.  Figure 6). The species are divided into five groups which reveal the complex integration between uses, different supply strategies, biogeographical origins, and gathering environments. The medicinal and edible species are found to be clearly separated, showing the absence of use complementation. One group is made up of medicinal species, spatially separated from cultivated species. Another group consists of edible species, close to the group of exotic plants which are obtained mainly through purchase and/or barter. Finally, the group of native species can be seen, which are obtained mainly by gathering in forest, steppe, and Patagonian monte environments. It is evident, then, that there is compartmentalisation not only of medicinal-edible use but also of the different strategies that increase the diversity of species and uses, which at the same time reflects the importance of this family to local populations.

## 4. Conclusions

This preliminary study reveals the current high level of usefulness of Fabaceae species in Argentine and Chilean Patagonia and the significant role they play as a food and/or medicinal resource. The bibliographical analysis has shown that the cultural practices associated with their use in the region, their recipes, and their symbolic value have been little studied, a subject which should be explored in more depth in the future.

At the present time, the different cultural enclaves in Patagonia use both native and exotic Fabaceae, indicating the hybridisation of knowledge and practices, a process which started with European colonisation of the region and continues due to the influence of the communications media. This process also reflects the great link that exists between the environmental changes in the surroundings, which, although we do not know whether they represent the cause or effect, demonstrates a direct connection between local populations and plants. The usefulness of this family is made more evident if the great richness of exotic species obtained through purchase, barter, or cultivation is considered. Different supply strategies are added to gathering practices, doubling the availability of Fabaceae in the region, and clearly showing its cultural value. This wealth of elements and practices indicates that due to their outstanding characteristics, the prominence of plants belonging to the Fabaceae family goes beyond ecology, since they also have an impact on socioeconomic and global society terms, which makes them abundant resources in shops and exchange networks. They also receive considerable publicity from various actors in society.

According to our bibliographical revision, there is little complementary medicinal-edible use of these plants. The question we must ask is whether this could be due to cultural patterns of knowledge loss regarding the varied uses of the species [4, 77, 86], to greater emphasis on their edible significance, to a singular, but also heterogeneous battery of phytochemicals in the group, or to limitations in the ethnobotanical study itself. Nevertheless, this work highlights the importance of considering the complete set of sociocultural and ecological factors that affect the use of any botanical group, thus establishing the different circuits involved in its use.

Finally, despite the contribution of bibliographical analyses to a general panoramic view of resource use, it should be noted that they are limited in that they offer “snapshots” of specific moments and depend on the varied visions of their respective authors. As proposed by Campbell and Luckert [74], resources are used in ways that fluctuate with time, in response to socioenvironmental changes experienced by human populations. For this reason, we emphasize the importance of employing a variety of research techniques in modern populations which will allow us to determine more accurately the cultural importance of the species used, and how this relates to the availability of plants in the environment. At the same time, we must be aware of other supply paths, with resources obtained from today's global society which play a significant role in the customs and habits of the people.

## Figures and Tables

**Table 1:** Edible and medicinal Fabaceae plants cited by 27 ethnobotanical sources of Patagonia. Fa: farming; P-B: purchase-barter; G: gathering; M: monte; S: steppe; F: forest; E: edible; Me: medicinal.

Species	Vernacular names	Origin	Strategies (ecological environments)	Uses	Growth habits
*Acacia aroma* Gillies ex Hook. & Arn.	tusca	Exotic	Fa; P-B	E, Me	Shrub
*Acacia caven *(Mol.) Mol.	huayun, cawén, caven, espino	Native	G (M); P-B	E, Me	Tree
*Adesmia boronioides* Hook. f.	paramela, té silvestre, yerba carmelita, éter, yagneu, lonkotrevo	Native	G (F, S, M)	Me	Shrub
*Adesmia emarginata* Clos	paramela	Native	G (F, S)	Me	Shrub
*Adesmia lotoides* Hook. f.	kiárksh	Native	G (F, S, M)	E	Herb
*Adesmia volckmannii *Phil.	mamül choike	Native	G (F, S, M)	Me	Shrub
*Anarthrophyllum *sp.	neneo macho, rülinlawen	Native	G (S, M)	Me	Shrub
*Arachis hypogaea* L.	maní	Exotic	P-B	E	Herb
*Astragalus garbancillo *Cav.	garbanzo, garbancillo	Exotic	P-B	Me	Herb
*Bahuinia forficata *Link	lawén-huiguln, pezuña de vaca, pata de vaca, pezuña de buey	Exotic	P-B	Me	Tree
*Caesalpinia paraguariensis *(D. Parodi) Burkart	guayacán	Exotic	P-B	Me	Tree
*Caesalpinia spinosa* (Molina) Kuntze	tara	Exotic	P-B	Me	Tree
*Cercidium praecox *(Ruiz & Pav. ex Hook.) Harms	brea, chañar	Native	G (M)	Me	Shrub
*Cicer arietinum* L.	garbanzo	Exotic	P-B	E, Me	Herb
*Cytisus scoparius *(L.) Link	retama, retama negra	Exotic	G (F); Fa	E, Me	Shrub
*Erythrina crista-galli* L.	ceibo	Exotic	P-B	Me	Tree
*Geoffraea decorticans *(Gillies ex Hook. & Arn.) Burkart	chañar, chical, chucal	Native	G (M)	E, Me	Tree
*Glycine max* (L.) Merr.	soja	Exotic	P-B	E, Me	Herb
*Glycyrrhiza astragalina* Gillies ex Hook. & Arn.	regaliz	Native	G (S)	Me	Shrub
*Hoffmannseggia erecta* Phil.		Native	G (M)	E	Herb
*Inga *sp.	pacay, guaba	Exotic	P-B	E	Tree
*Lathyrus magellanicus* Lam.	alvergilla	Native	G (F)	E, Me	Herb
*Lens culinaris *Medik. c1	lenteja turca	Exotic	P-B	E	Herb
*Lens culinaris* Medik. c2	lentejón	Exotic	P-B	E	Herb
*Lens culinaris *Medik. c3	lenteja	Exotic	P-B	E	Herb
*Lupinus albus* L.	lupino	Exotic	P-B	E, Me	Herb
*Lupinus *sp.	lupino, chocho	Exotic	G (F); Fa	E	Herb
*Medicago lupulina* L.	lupulina	Exotic	G (F, S, M)	E	Herb
*Medicago sativa* L.	alfalfa, alfa	Exotic	G (F, S, M); Fa	E, Me	Herb
*Melilotus albus *Desr.	meliloto, trébol de bokhara	Exotic	G (F, S, M)	E	Herb
*Melilotus indicus *(L.) All.	trébol de olor, trebillo, trévül	Exotic	G (F, S, M)	Me	Herb
*Melilotus officinalis *(L.) Lam.	meliloto	Exotic	G (F, S, M)	Me	Herb
*Otholobium glandulosum* (L.) J. W. Grimes	Külen, trafilawen	Native	G (F); P-B	E, Me	Tree
*Phaseolus coccineus *L.	poroto chileno, poroto colorado	Exotic	Fa; P-B	E	Herb
*Phaseolus lunatus *L. c1	poroto pallar, ayayo, ailladito	Exotic	Fa; P-B	E	Herb
*Phaseolus lunatus* L. c2	poroto manteca	Exotic	P-B	E	Herb
*Phaseolus vulgaris* L. c1	frijol, poroto, purutu, dengüll, cüllhui	Exotic	Fa; P-B	E	Herb
*Phaseolus vulgaris* L. c2	poroto negro	Exotic	P-B	E	Herb
*Phaseolus vulgaris* L. c3	poroto colorado	Exotic	P-B	E	Herb
*Phaseolus vulgaris *L. c4	chaucha	Exotic	P-B	E	Herb
*Phaseolus vulgaris *L. c5	poroto alubia	Exotic	P-B	E	Herb
*Pisum sativum* L.	arveja, alverja	Exotic	Fa; P-B	E, Me	Herb
*Prosopis alba* Griseb.	tacu, huilca, huancu, algarrobo	Exotic	P-B	E	Tree
*Prosopis alpataco *Phil.	alpataco, soil mamül	Native	G (M)	E	Shrub
*Prosopis chilensis* (Molina) Stuntz emend. Burkart	algarrobo blanco	Exotic	P-B	E	Tree
*Prosopis denudans *Benth.	algarrobo	Native	G (M)	E	Shrub
*Prosopis flexuosa* DC.	algarrobo dulce, yoiwitru	Native	G (M)	E	Shrub
*Prosopis rubiflora *Hassl.	vinal	Exotic	P-B	Me	Shrub
*Prosopis strombulifera* (Lam.) Benth.	retortuño, pata de loro, chowel	Native	G (M)	Me	Shrub
*Pterocarpus santalinoides* L'Hér. ex DC.	sándalo	Exotic	P-B	Me	Tree
*Robinia pseudoacacia* L.	acacia blanca	Exotic	G (F); Fa	E	Tree
*Senna alexandrina* Mill.	sen	Exotic	P-B	Me	Shrub
*Senna stipulacea* (Aiton) H.S. Irwin & Barneby	quebracho, mayu, traftrafén	Native	G (F)	Me	Shrub
*Sophora cassioides* (Phil.) Sparre	pëlú	Native	G (F)	Me	Tree
*Sophora macrocarpa *Sm.	mayu, pëlúpëlú	Native	G (F)	Me	Tree
*Sophora microphylla* Aiton	pëlú	Native	G (F)	Me	Tree
*Tamarindus indica* L.	tamarindo	Exotic	Fa; P-B	E, Me	Tree
*Trifolium pratense *L.	trébol rojo	Exotic	G (F, S, M)	E	Herb
*Trifolium repens *L.	trébol blanco	Exotic	G (F, S, M); Fa	E	Herb
*Trigonella foenum-graecum* L.	fenogreco	Exotic	P-B	E, Me	Herb
*Vicia faba* L.	haba	Exotic	Fa; P-B	E, Me	Herb
*Vigna angularis* (Willd.) Ohwi & H.Ohashi	aduki	Exotic	P-B	E	Herb
*Vigna radiata *(L.) R. Wilczek	poroto mung	Exotic	P-B	E	Herb
